# Dilemmas when talking about Huntington's disease: A qualitative study of offspring and caregiver experiences in Norway

**DOI:** 10.1002/jgc4.1610

**Published:** 2022-07-29

**Authors:** Siri Kjoelaas, Tine K. Jensen, Kristin B. Feragen

**Affiliations:** ^1^ Centre for Rare Disorders Oslo University Hospital HF Rikshospitalet Oslo Norway; ^2^ Department of Psychology University of Oslo Oslo Norway; ^3^ Norwegian Centre for Violence and Traumatic Stress Studies Oslo Norway

**Keywords:** caregivers, communication, family, Huntington's disease, offspring

## Abstract

Research provides a compelling list of reasons why offspring should be included in honest conversations about disease when the disease affects their caregivers. Despite this, we lack in‐depth knowledge about how families affected by the severe and complex genetic condition Huntington's disease (HD) experience talking about the many aspects of how this disease affects their lives. This study aimed to provide an in‐depth exploration of how offspring with a caregiver with HD and caregivers with a partner with HD experienced talking about disease throughout childhood. Thematic analysis was conducted with semistructured interviews of both caregivers (*n* = 14) and offspring (*n* = 36) from families affected by HD, reflecting both current and past experiences. In addition to highlighting the many needs offspring have for knowledge and conversation about the disease with their caregivers, our findings also show that a variety of dilemmas can follow these conversations, including when to talk, what to say, how often HD should be talked about on a day‐to‐day basis, and whether to share disease‐related information with others outside the family. The findings show the complexity of talking with offspring about HD. A difficult task for both offspring and caregivers seemed to be finding out how to balance the many dilemmas that arise in conversations and how to use dialogue to best help offspring adapt and cope with the many challenges that can come with HD. The findings can assist health care professionals, such as genetic counselors, prepare, and guide families affected by HD in the many and complex conversations that arise about the disease, in turn helping offspring adjust and cope with their current lives or future lives affected by HD.

**What is known about this topic**

- Huntington's disease is a neurodegenerative genetic disease that, in affected families, presents major challenges for offspring.
- The many benefits that come with informing and including offspring in honest conversations about the disease when it affects their caregivers are well‐established.

**What this paper adds to the topic**

- This paper adds a unique and in‐depth presentation of both the caregiver's and offspring's perspectives regarding the many aspects of talking about the complex genetic condition of Huntington's disease with offspring.
- The findings suggest that talking with offspring about Huntington's disease extends beyond informing them about genetic risk, and that many dilemmas arise through these conversations.
- Caregivers may need help to navigate through the many dilemmas that arise in conversations with offspring to ensure that offspring in families with Huntington's disease have the best opportunity to adapt and cope.

## 1 INTRODUCTION

Severe genetic disease does not only affect one individual; it can affect a whole family, particularly the offspring. To help offspring adapt to and cope with having a caregiver who suffers from severe disease, research strongly suggests that they should be included in honest conversations about their life situation (Dalton et al., 2019). However, caregivers often find it hard to talk about the disease with their children and struggle with whether, how, and when to have these talks, and with managing the emotional responses that follow (e.g., Dalton et al., 2019; Metcalfe et al., 2008). This also seems true when talking with offspring about genetic risk in families affected by severe and complex heritable conditions, such as Huntington's disease (HD; e.g., Gaff et al., 2007; Metcalfe et al., 2008; Rowland & Metcalfe, 2013). Although caregivers may need help from health care professionals, such as genetic counselors, to successfully manage conversations with their offspring, we lack in‐depth knowledge about how both caregivers and offspring experience the many aspects of the conversations that can come with having a caregiver with HD and how talking about the disease helps or hinders offspring's adaptation and coping.

### 1.1 Huntington's disease

A neurodegenerative genetic disease, HD causes a progressive and fundamental deterioration of an individual's physical, cognitive, and psychological functioning (Roos, 2010). Because the typical age of onset for HD is between 30 and 50 years, it is also a time in life when many have caretaking responsibilities for offspring when their symptoms first occur (Roos, 2010). HD has a 50% risk of genetic transmission, meaning that offspring are at risk of having inherited the gene and developing the disease themselves. The slow and long‐lasting progression also means that both the individual with HD, and their family will live with the disease for an extended time, an average of 17–20 years, before it ultimately leads to death (Roos, 2010). As the disease progresses through multiple stages, the impact on offspring can be even longer. One of these stages includes having a caregiver who is at risk or presymptomatic, with progressing changes that develop slowly and symptoms that are often present years before a diagnosis is given (Duff et al., 2007). Such symptoms include, but are not limited to, involuntary movements, personality changes, aggression, and apathy (Roos, 2010). Many of these symptoms change the functions needed for caregivers to appropriately understand and respond to their offspring. For instance, cognitive symptoms interfere with the individual's emotional recognition, which may hinder a caregiver's abilities to appropriately read, recognize, and respond to their child's emotions (Watson et al., 2021).

### 1.2 Growing up with a caregiver with HD

A relatively small but significant body of research has reported on the lived experiences of young people in families with HD, showing that they can grow up with many challenges and stressors as a result of their caregiver's disease. Although growing up with a caregiver with any disease can be a major challenge, HD may present an additional range of unique and complex issues. For instance, HD can break down family systems and compromise the support young people get from both caregivers, effectively leaving many isolated (Forrest Keenan et al., 2007; Kjoelaas et al., 2021; Vamos et al., 2007). One study found that compared with young people who have a caregiver with another inheritable condition—BRCA1/2 breast/ovarian cancer—those with a caregiver with HD reported significantly more negative life events (van der Meer et al., 2012). The profound challenges posed in the lives of young people in families with HD include, but are not limited to, overwhelming responsibilities as carers (Kavanaugh, 2014; Williams et al., 2009), insecurities about their own future because of the inheritability of the disease (Lewit‐Mendes et al., 2018), and disrupted family functioning (Forrest Keenan et al., 2007), including parental dysfunction (Vamos et al., 2007) and exposure to a range of adverse experiences, such as complicated grief, unpredictability, violence, aggression, substance abuse, and suicide (Kjoelaas et al., 2021; van der Meer et al., 2012).

### 1.3 Talking about Huntington's disease

Although there are available resources for support for family members of someone with HD, such as the Huntington's Disease Youth Organization (HDYO), informing and talking with offspring about the disease is usually seen as the responsibility of family members (Forrest et al., 2003). However, past research looking at families' experiences with informing offspring about genetic risk has indicated that talking about HD may not be an easy or straightforward task. Therefore, those at risk of inheriting HD learn about their family's history of disease in different ways and at different times in their lives (Etchegary, 2006; Forrest et al., 2009; Metcalfe et al., 2011). When it comes to telling or not telling offspring about HD, caregivers seem to be faced with many challenges, including whether to tell, who should tell, when to tell, and how to tell (e.g., Forrest et al., 2003; Stuttgen et al., 2021). This could partly be because of an inherent dilemma caregivers face: They must choose between the obligation to convey valuable information about genetic risk and their wish to protect their children from information that could cause them to harm or worry (Gaff et al., 2007). Other studies have suggested that offspring generally want to learn about HD and its genetic risk at a young age (Forrest Keenan et al., 2007; Holt, 2006; Stuttgen et al., 2021) and have emphasized the importance of having honest conversations about HD (Sparbel et al., 2008). However, research also suggests that receiving information about HD and its associated risks can be difficult for offspring, leaving them feeling overwhelmed by the information, or feeling like they were told the wrong way (Forrest et al., 2009). These and similar studies have highlighted the distressing and often conflicting processes involved in conversations about the genetic risk that families with HD go through. So far, the main focus of research on talking with offspring about HD has been on informing or being informed about genetic risk and genetic testing. Therefore, we continue to lack in‐depth knowledge about the many conversations families could have related to all other aspects of the consequences of the disease and how these conversations are experienced by both the offspring and caregivers.

### 1.4 The importance of conversations

The many stressors and challenges young people in families with HD experience provide strong reasons to believe that there is a range of aspects regarding the disease that offspring may want to know about and discuss with their caregivers. The conversations caregivers have with their children will play a major role in any child's development but are perhaps particularly important when trying to help children make meaning out of challenging situations (e.g., Ellis et al., 2017; Morris et al., 2016). For instance, children in any family will develop an understanding of themselves and others based on their social experiences (Feldman, 1992). Through verbal and nonverbal interactions with those who are close to them, children build ideas about how the world works by actively trying to make sense of situations, events, and their surroundings (Bruner, 1990). Understanding their surroundings is perhaps particularly important for children who are faced with changes and difficulties, such as their caregiver having HD, as they try to restore a sense of control and predictability by attempting to understand the changes that are occurring. In these situations, conversations within the family help children form an understanding of existential concepts, such as life and death (Keeley, 2016). Research seems to support this notion, providing compelling evidence for why offspring should be included in continuous conversations when their caregivers are affected by the severe disease (Dalton et al., 2019; Rowland & Metcalfe, 2013). For instance, one meta‐synthesis of research on family communication about genetic risk concluded that for coping, offspring are helped by having conversations that will check their understanding, provide explanations, and help with ongoing feelings and concerns (Metcalfe et al., 2008). For other types of disease, such as cancer, conversations between caregivers and offspring have been established as a particularly important tool for support and coping in everyday life (Ellis et al., 2017; Morris et al., 2016; Stone et al., 2012) and for decreasing their risk of a range of negative psychological outcomes, including anxiety and depression (Dalton et al., 2019).

### 1.5 Study aim

Given the importance placed on talking with children about their caregiver's disease in general, there is a need to understand more about conversations between caregivers and offspring when it comes to HD, along with how these conversations help or hinder offspring's coping and adaptation. Because the nature of HD is complex and presents a variety of unique and prolonged stressors for offspring, talking about the disease could be particularly challenging for both the offspring and caregivers. Therefore, families might need help and guidance from health care professionals to effectively prepare for and navigate through the many topics of conversations about the disease so that the offspring will adapt and cope. To accomplish this, those in contact with families affected by HD, such as genetic counselors, need more knowledge about the range and complexity of conversations families possibly have with offspring about HD. To address this, the current study aimed to provide an in‐depth exploration of both caregivers' and offspring's perspectives on talking about the many aspects of HD throughout childhood.

## 2 METHODS

### 2.1 Study participants and recruitment

Qualitative data were collected as part of a larger study that has the overall aim of exploring several topics related to experiences of growing up with a parent with HD, here in a Norwegian context. In the present study, we analyzed interviews of offspring with a caregiver affected by HD (collected from 04/2018–09/2018) and caregivers who have children with a partner with HD (collected from 07/2019–08/2020). Anyone in Norway over the age of 12 years who had current or previous experiences of growing up with a caregiver with HD and anyone who has/had children with a partner with HD were invited to participate. Information was distributed verbally and through information sheets in different settings where individuals in families affected by HD could be reached. These locations included educational courses for families affected by HD, counseling services at Oslo and Haukeland University Hospitals and at St. Olavs Hospital, the Norwegian Association for Huntington's Disease, along with online settings. Information sheets outlined the study's purpose, provided information about the interview topics, and included the main researcher's name and contact information. Table 1 summarizes the participants' demographic information. In response to the formal and informal invitations, 36 offspring and 14 caregivers gave their written consent to participate and were included in the analysis (*N* = 50). Offspring ranged in age from 13 to 65 years (M_age_ = 36.6 years) and caregivers from 42 to 67 years (M_age_ = 54.9 years). The participants reflect a broad spectrum of family life stages, patterns of progression of HD, and formal health care and support systems.

**TABLE 1:** Demographic characteristics of the participants (*N* = 50)

Offspring (*N* = 36)	
Age Group of Offspring	
Teenager (13–18 years)	7
Young adult (19–35 years)	10
Adult (36–65 years)	19
Gender	
Female	26
Male	10
Caregiver with HD	
Mother	19
Father	17
Caregivers (*N* = 14)	
Age Group of Children	
School‐aged children (0–12 years)	3
Teenagers (13–18 years)	4
Young adults (19–35 years)	5
Adults 36–65 years	2
Gender	
Female	11
Male	3

### 2.2 Data collection

Separate interview guides for the offspring and caregivers were created based on similar topics, relevant literature, and feedback from clinical experts and a group of user representatives. Individual semistructured interviews were conducted, focusing on both the challenges and protective factors. Broad topics included offspring and caregiver's experiences of growing up in a family with HD, openness about the disease, and experiences of support. For the purpose of the current study, we defined childhood as the phase of life between 1 and 12 years and adolescence as the phase between 13 and 19 years. Table 2 displays the interview topics and sample questions. On average, the interviews lasted 60 min (range: 27–90 min). Face‐to‐face interviews were generally preferred (*n* = 46); however, a few participants preferred telephone interviews (*n* = 4). The interviews were conducted at the Centre for Rare Disorders at Oslo University Hospital, in other locations outside the home, or in the homes of a few participants. The location of the interviews was based on the preference of our participants. Because many of the participants had challenging home lives with partners or caregivers with HD or felt it was easier to talk in the privacy of our outpatient hospital department, most of the participants preferred to be interviewed outside the setting of their home. Counselors from the Centre for Rare Disorders were involved in planning the study, but they were not involved in data analyses because of their involvement with potential participants. Several researchers with formal training in addressing sensitive topics conducted the interviews, including postgraduate clinical psychology students, a clinical psychologist, and a genetic counselor. The students had no previous experience with HD, whereas the health professionals had previous research experience with the disease. All the interviewers had or had received formal training in qualitative methods before conducting the interviews. The project manager performed sample tests on the correspondence between the recorded interviews and transcripts to ensure accuracy between the transcripts produced by different members of the research team. The interviewers had no previous familiarity with the study participants. Supervision regarding interview techniques was provided, if needed. The project manager (an experienced licensed clinical psychologist) participated in at least two interviews conducted by novice researchers to ensure the reliability and consistency of all the interviewers' practices.

**TABLE 2:** Main topics and sample items applied in the semistructured interview guide

Interview topic	Sample question offspring	Sample question caregiver
Background information	Please describe the family you grew up in?	Please describe the family your children grew up in?
Childhood experience	How did your parents' disease affect your family?	How did your children experience the disease?
	How has growing up with a parent with HD affected you?	How do you think growing up with a parent with HD affected your child/ children?
Disease‐ and self‐disclosure	How did you first learn about HD?	How did your children first learn about HD?
	What do you feel are the benefits and disadvantages of disclosing information about HD?	What do you feel are the benefits and disadvantages of disclosing information about HD to children?
	What were your experiences with asking about HD if you had questions or concerns?	What information and conversations about HD did you feel like your children needed?
Resources and support	What sources of support did you have growing up?	What sources of support did your children have growing up?
	What support could you have needed that you did not get?	What support could your children have needed that they did not get?

### 2.3 Data analysis

The last author, who is also the project manager, performed sample tests on the correspondence between the recorded interviews and transcripts to ensure accuracy between the transcripts produced by different members of the research team. The analysis was guided by Braun and Clarke's thematic analysis (Braun & Clarke, 2006, 2019). During the analysis process, all authors became familiar with the data by reading and rereading the interview transcripts. The first and second authors generated initial codes by isolating phrases, sentences, and paragraphs, generating a list of codes representing every transcript. The lists of codes were collated to search for themes according to the similarities between them. During this step, we noted how the participants consistently spoke about how they had prepared, communicated, and experienced the topic of talking about HD; this became the focus of the analysis. As we continued to analyze the topic, we found that the participants described a multitude of difficult choices or *dilemmas* that did not necessarily have an obvious or preferable solution. These dilemmas were categorized and used as the overarching framework. It may be important to note that although several participants reflected on the actual dilemmas presented in their interviews, this was not the case for all the caregivers or offspring. Rather, the dilemmas used as the framework in the analysis were found within and between the participants. Themes were reviewed against the data and discussed between the three authors until full agreement had been reached. Table 3 summarizes the main themes and corresponding subthemes.

**TABLE 3:** Overview of themes and subthemes

Main theme	Subtheme
Too soon or too late?	Maturity or adaptability?
	My timing or your timing?
Too much or too little?	Honesty or caution?
	Too often or not enough?
To share or not to share?	Help or stigma?
	My choice or your choice?

In the presentation of the findings, the frequency labels *general*, *typical* and *variant*, as suggested by Hill et al. (2005), are used to indicate the degree of representativeness across individual cases. The topics or themes that were general in the sense that they applied to all but one participant are referred to in the text as *all participants*. Topics were considered typical if they applied to more than half of the cases and are referred to as *most participants*. Topics were variant if they were represented in less than half of the sample but in more than two cases; they are referred to in the text as *some participants*. Quotes that represent the themes and subthemes were selected and translated from the original language into English. The participants were given pseudonyms to preserve their anonymity.

### 2.4 Reflexivity

Reflexivity was important throughout the research process. The first and last authors have experience with HD as researchers. Theoretical knowledge about HD was seen as beneficial during data collection and analysis because it helped to recognize features in the participants' experiences specific to HD. However, this knowledge could also create biased interpretations of the interviews. To enhance validity and counteract group thinking and researcher bias, the first and last authors formed the primary analytic team. The second author, who has extensive experience working with developmental psychology in clinical and research settings but no previous experience or knowledge of HD, analyzed the interviews independently and served as a discussant. Therefore, we were able to benefit from the different positions we had and question any theoretical and professional interpretations made. We encouraged reflection on our different positions and experiences. Consensus was obtained after repeated rounds of independent reading, sharing of notes and open discussions, and then rereading and rediscussing of the interviews.

### 2.5 Ethical considerations

The participants were informed about the study and their right to withdraw at any time before giving their written consent. The confidentiality of participant information was ensured throughout the research process. Caregiver consent was obtained from the participants under the legal age for health consent (16 years in Norway). Because of the sensitivity of the topics discussed during the interviews, relevant referrals or subsequent follow‐ups were arranged, if necessary. Five participants wished to receive follow‐up after the interviews and were referred to a clinical psychologist or counselor working with families affected by HD.

## 3 RESULTS

Despite variations in how and when HD affected the families of the participants when it came to talking about the disease, there seemed to be a large overlap in the main topics addressed by both the offspring and caregivers. In general, having conversations with offspring about HD seems to be a complex task that could last throughout childhood. Common to most families was the lack of available guidance from health care or support services when navigating the dilemmas raised by their conversations. The dilemmas that the families had been faced with were categorized and used as the overarching framework to present the participants' experiences of the different aspects of talking with offspring about HD. These categories included three main themes: *Too soon or too late? Too much or too little?* and *To share or not to share?* and their corresponding subthemes.

### 3.1 Too soon or too late?

Taken together, both the caregivers and offspring brought forth several dilemmas related to *when* offspring should be told about HD.

#### 3.1.1 Maturity or adaptability?

A central dilemma relating to *when* offspring should be told about HD is whether they would benefit from waiting to learn about the disease until they were mature enough to understand its complexity or if it was best to talk to offspring at a young age so that they could adapt to and integrate the knowledge as they grew older.

In the current study, the caregivers argued for both options. When making this decision, all the caregivers seemingly weighed the risks and benefits of the different options before choosing the option they felt would best protect their child. Some wanted to wait until their children were older because they were concerned that talking with them about HD at an early age would lead to fear and worry about the future and leave their children in a situation that could be difficult to cope with. One father, who had not yet told his school‐aged son that the disease affecting his mother was HD, described why:

It comes down to the fact that it's a disease he might start thinking he could have. I want him to wait to have to do that until he is done being a kid. But then again, he could figure it out on his own. I can't control that. (Jeffrey)

As this father also suggested, the other option caregivers had chosen was to tell as early as possible, based on the fear that by waiting too long, their children could get the information elsewhere and possibly be misinformed and confused.

Many of the offspring seemed to understand the dilemma their caregivers faced when choosing between telling earlier or later. One teenager, who had known that his father had HD for as long as he could remember, reflected on the choice his mother had made:

It's a really difficult balance. You either wait until they are old enough to understand, but then, you have to wait for a long time. Or you tell them right away, but then, they might not understand and might worry unnecessarily. (Michael, teenager with a father with HD)

As Michael has indicated, there was not necessarily a ‘good’ time to learn that his caregiver had HD. Nevertheless, most offspring seemed to strongly favor being told as early as possible:

Children will adapt to things; that's the way it is. You learn how the world works and that this is a part of your world. (…) So yes, I believe it was right that I was told as early as I was. (Nicole, adult with a father with HD)

The benefits of being told in early childhood, as Nicole suggested, include adapting to this knowledge as one grows older. Learning about HD at an early age also seems to have spared the offspring much of the shock that those who were told in adolescence and adulthood often described:

I would have wanted to grow up knowing that my mom was going to get sick instead of them lying and it coming as a shock.(Mia, teenager with a mother with HD)

Not being told until adolescence or adulthood, but only if the disease had been known to their caregivers, often made the offspring feel like they had been lied to and had the possibility of severely disrupting their relationship with and trust in their caregivers. In some instances, the offspring said that they had accepted and understood why they had not learned about the disease earlier. For instance, HD had not been known in some families, and the caregivers did not have this information to share with their children at a younger age. Also, knowing that their caregivers had withheld information from them in an effort to protect them seemed to help the offspring accept why they had not been told sooner. However, learning about HD in adolescence and adulthood came with a range of other complications, regardless of the reasons why their caregivers had not talked about HD at an earlier age. For instance, the new information seemed to collide with already challenging tasks at the time they were told, such as planning a future, engaging in romantic relationships, or having children (especially regarding the possibility of having passed the disease on to their offspring). For some, it also seemed to have been the catalyst for severe reactions, including depression and suicidal thoughts.

#### 3.1.2 My timing or your timing?

Another central dilemma related to *when* offspring are told about HD is the decision that nonaffected caregivers have to make between the needs of their children and those of their partner with HD.

A few caregivers had anticipated that this might become a dilemma, often in collaboration with their partner with HD before symptoms occurred, and had planned decisions about the time and event before talking with their children about the disease. However, putting their children's needs first was not always an easy task. Instead, because of a lack of insight into the progression of the disease, their partners with HD had not wanted or did not feel the need to tell their children. Therefore, the nonaffected caregivers had been caught in the dilemma of choosing between their own wish to tell their children and their partner's need to deny the subject. For many, this included long negotiations between caregivers, often at the expense of telling their children, as described by this father:

I knew for years that she [partner with HD] would get the disease, but I couldn't tell the kids because that would go against what she wanted. Of course, I could have done that, but I didn't feel it was right. (…) And what's right then? I don't know … It will be a challenge as long as the one involved doesn't want to tell. (Gregory, father of young adult children)

The question of whose needs should be prioritized when it comes to children learning about HD did not seem to be experienced as a dilemma for most of the offspring. Instead, they consistently emphasized how they needed or would have needed to learn about HD before symptoms occurred. They were sensitive to changes brought on by the disease and shared how not understanding the slow and subtle alterations had negatively affected them long before their caregivers thought they were noticeable and needed to be explained. Lacking an explanation, many had blamed themselves and were consequently ill‐equipped to mitigate the impact HD could have on their future. The offspring also highlighted the importance of being told in planned, rather than spontaneous, events and the importance of caregivers coming forth as emotionally stable and consistent. However, for many, this had not been the case, and they had experiences of seemingly normal events that transitioned into being told about the disease without forewarning. Common to the spontaneous events was that seemed to leave the offspring with the impression that it was their caregiver's needs, and not their own, that had been the priority. Conversations that did not come across as planned seemed to increase feelings of shock, as in the case of this teenager, who spoke about the recent event in which he learned that his father had HD:

It was so strange how he told us about it; he was probably out of it himself. He just walked up to my mom and said that he got the test results and that he had HD. Smiling and crying, like he had just won a football game. That really pissed me off. (Jason)

Other offspring described how they had only learned about the disease as a premise for the next steps they were to take in life, such as choosing their education or entering serious romantic relationships.

### 3.2 Too much or too little?

In addition to *when* offspring should be told about HD, there also seemed to be a range of dilemmas related to *what* to say and *how often* they needed to talk about the disease on a day‐to‐day basis.

#### 3.2.1 Honesty or caution?

A central dilemma in terms of *what* to say when talking with offspring about HD was whether they should be told about the disease with complete honesty or whether caregivers should be more careful when conveying this message.

Being left with the power to decide what information was important for their children to have had not been an easy task for caregivers. On the one hand, some feared the consequences of being completely honest and had been cautious about any information they provided:

I think it's ok to tell the kids about the disease so that they will know what it is, but I feel that you should be really careful about saying too much. Because my son started thinking about it a lot, it really made him worry. (Barbara, mother to adult children)

On the other hand, other the caregivers wanted to be as honest as possible because they feared the consequences of providing too little information. However, despite wanting to be honest, it still seemed difficult to determine what to say to children at different ages and how to ensure that their children understood the information they received:

I do wonder how informed they are. At the same time, we have all these magazines available so that they can read when they want and figure it out. I think it's difficult to force‐feed them with information when they are not asking for it. But what Huntington's looks like in their minds? I really don't know. (Jill, mother of teenage children)

Other caregivers had been left with questions about who should be in charge of asking for more information if needed, fearing that they would create worry by informing too much, but finding it difficult to ask their children what they understood and what information they needed.

Taken together, the offspring also seemed conflicted between honesty and caution:

I want to know, but at the same time, I really don't want to know. Very difficult. I told my dad that unless it's incredibly relevant, I don't want to hear about it at all. (Kyle, teenager with a father with HD)

Having been shocked when learning about the disease, some were unsure if children should be told about the more detrimental aspects of HD from a young age:

When I was a teenager, I was told everything at once and that it was inheritable. It was a lot; it really was. So I am delighted to hear that people are getting better at talking with their children because I didn't really have that. (Tina, adult with a mother with HD)

At the same time, not being sufficiently informed, receiving inaccurate or misleading information, or feeling rejected by caregivers when asking for information seemed to leave the offspring without an understanding of the disease and its consequences. Others felt that they had been provided with either too much information or insufficient information at a young age, without any follow‐up to information as they grew older. Although the offspring seemed unsure about what information they wanted, they provided clear suggestions on what they did not want. For instance, they consistently stressed how important it was for children to not be lied to: ‘Don't lie to children about HD. Nothing good will come of that. Both options will be difficult for them, but it's better to just be honest' (Kourtney, adult with a mother with HD). To accommodate the seemingly conflicting needs of the offspring for honesty and caution, being informed via a continuous process rather than a surprising, one‐time event seemed particularly important.

#### 3.2.2 Too often or not enough?

After the offspring had been told about HD, another dilemma seemed to arise: How often do they need to talk about the disease on a day‐to‐day basis?

The caregivers had seemingly been faced with the dilemma of finding out how much their children needed to talk and how to support those struggling to cope with the disease. After initially telling their children, some had avoided addressing the subject because they feared what emotions the conversations could trigger and worried that if they mentioned HD too often, their children would not be able to escape from the disease and live a normal life without excessive worry:

It can't be all about Huntington's every day. I could see in my daughter how talking about it bought her down and all the time we spent trying to get her back up …. Many people in our family have committed suicide because of this, and her thoughts were there, too …. So you should tread carefully. That's my opinion. (Barbara, mother to adult children)

In contrast, other caregivers wanted to ensure that their children were not repressing feelings and aimed to model openness by addressing the day‐to‐day difficulties:

When something happens, I will talk with the kids right away. I tell them they know how their dad is …. And usually they will answer, ‘Yes Mom, we know it's Huntington's. You don't have to talk about it right now, Mom. It's enough, we know'. (Christina, mother to school‐aged children)

As this mother suggests, when it comes to talking about the disease, the caregiver's and offspring's needs do not always match. Some were genuinely worried about how their children were coping with HD and the difficulties they could see that it was creating in their developing lives; they had wanted to use conversation as a tool to help their children but had felt rejected in their attempts because their children felt that any mention of the subject was too much. Feeling powerless over their lack of ability to reach out and provide support, these caregivers had to go to great lengths to ensure that their children felt they had someone available to them if they needed to talk about their situation. To accomplish this, they had to be creative, use indirect ways of communication, and often endure difficult conversations with their children, particularly during the teenage years.

The offspring seemed to have a variety of individual needs when it came to talking about HD on a day‐to‐day basis. On the one hand, several said that they consistently rejected their caregiver's attempts to talk about the disease; they struggled with expressing and understanding their emotions related to the disease, and wanted to refrain from the subject to maintain a sense of normalcy in their lives or wanted to protect their caregivers from their own thoughts and worries about HD:

My dad and I do talk quite a lot. But there are things I don't want to tell him because I feel that he has a lot on him already. So I kind of limit how much I say because of that. (Penelope, teenager with a mother with HD)

However, not wanting or feeling the need to talk about HD seemed to be reserved for those who felt that they had caregivers available to do so if they wanted. For most of the offspring, HD had not been addressed on a day‐to‐day basis, and they did not feel like they had a caregiver to whom they could talk. Consequently, they often longed for a caregiver who would talk to them about minor or major difficulties, finding that a lack of initiative or silence when it came to issues related to HD had severe consequences, including increased worry:

I think talking about it [HD] and involving everyone, regardless of age, is super important. (…) I can remember all the whispering and secrets, even when I was a teenager, giving me the impression that I wasn't included. It was not a good thing. And when you sense something is said and done, you'll create this version in your head that's a lot worse than the reality. So … to communicate, that is very important. (Brenda, adult with a mother with HD)

For Brenda, not talking about HD growing up had truly impacted her childhood because it left her wondering what was happening and created a feeling of not having much‐needed support.

### 3.3 To share or not to share?

In addition to the dilemmas faced when talking with offspring about HD within the family, the participants had also faced dilemmas related to whether they should tell others outside the family about the disease.

#### 3.3.1 Help or stigma?

A central dilemma was whether sharing information about HD with others would be of help or if it would increase the burden on the offspring.

For most of the caregivers, informing others, such as schools or other health care services, was seen as an opportunity for their children to be supported, but it was followed by a potential risk of social cost. Some feared that the curiosity or judgment of others could leave their children vulnerable to others' questions and concerns, and they felt they had to prepare their children for the reactions of others. One mother, for whom HD had come as a surprise when her children were teenagers and who had chosen to fully disclose information to everyone they knew, stated the following:

I had to go and tell my kids that if anyone asks any questions, you can choose if you want to answer them or not. Because I knew that people who wouldn't ask me might use them to get information. (Mary, mother to teenage children)

Other caregivers wished they had disclosed this information sooner, having seen that it could have had the potential to help their children.

The offspring shared both positive and negative experiences of talking with others outside their families about HD. The decision to tell others seemed to be based on certain premises of those with whom the information would be shared, such as talking with others who are trusted and understood, and/or who were in a similar situation. The benefits of talking with others included having friends who know what the family is going through, which made them feel less lonely and reduced negative social reactions from friends. However, the offspring also frequently encountered negative experiences when telling others. For instance, some had tried talking with others for support but did not feel that it had helped their situation. Others described what they perceived as stigmatizing experiences, such as having a teacher point them out in a class while lecturing about genetic diseases. Not receiving the support and understanding they needed when talking with others seemed to leave the offspring feeling rejected, hindering them from reaching out to others for help and support in the future.

Regardless of how the offspring felt about talking with others about HD, the heritability of the disease seemed to be a particular barrier. The offspring said that they had often been conscious about how this information could be interpreted and feared that their friends would shy away from them socially or that others would be looking for symptoms of HD in them:

Because of the possibility that I could develop HD, too, I was careful with what I told others. It's a little scary; they might see things they otherwise wouldn't see, and I could be treated differently. (Gina, adult with a father with HD)

Fear of the stigma associated with disclosing information about HD also extended beyond social interactions. For instance, the offspring feared that it could potentially be harmful to their futures, such as hindering them from obtaining promotions at work or insurance.

#### 3.3.2 My choice or your choice?

Another dilemma that the participants faced when deciding whether to tell others was who had or should have the right to decide to share on behalf of the offspring.

For the caregivers, this seemed to be a dilemma of whether to tell others, with or without the blessing of their children. Although the caregivers wanted to help their children by telling others, it could also be a violation of their children's privacy. This decision had not always been static but rather evolved with their child's age and needs. For instance, information that caregivers had confidently shared with others when their children were younger could have the potential to later interfere with their children's evolving need for independence and control as they grew older:

I am much more nuanced when it comes to sharing information than I used to be. It's something they have to be able to choose and that they are not obligated to do. And I'm terrible at it. I'll answer every time someone asks about inheritance and everything. And I think to myself, why do I have to do that? Can't they be allowed to decide if they want to answer these questions? Why do I need to hold that lecture? Not that there is anything they should be ashamed of but because it's their choice to make. (Jill, mother to teenage children)

Others also expressed how they did not want to overstep the boundaries set by their children and described how they had waited, ‘planting the seeds’ of possibilities and hoping that one day, their children would reach out for help and support.

Seen from the perspective of the offspring, being able to choose what others know about HD was related to a dilemma between maintaining control and autonomy and receiving help. Several offspring appeared to have quite strong opinions about sharing their caregiver's diagnosis with others. For instance, some offspring insisted that caregivers should not share any information on their behalf:

I got the feeling that I didn't have that control. One thing is what I chose to share; another thing is what I can't control. So in terms of HD and children, I would never have involved the school or friends or anyone. I would have kept my mouth shut and let the children make their own decisions. (Cheryl, adult with a mother with HD)

As Cheryl suggested, the offspring's need for control over what is shared on their behalf seems at times to collide with their caregivers' idea of what would help them. In contrast, other offspring had wanted to share information about their situation but had been hindered by their caregivers' or family's desire to maintain secrecy. Some of these offspring described how they deliberately crossed their caregivers by telling others about the disease and facing the possibility of alienation from their family. The offspring were also particularly aware that choosing to tell others about HD would provide information about other family members' genetic risk. This complicating factor often hindered their decision to disclose information about the disease and seek the support of others.

## 4 DISCUSSION

The current study provides an in‐depth exploration of both the caregivers' and offspring's perspectives on the many aspects of talking about HD. First, the findings suggest that offspring in families with HD have several general needs when it comes to talking about the disease with their caregivers. These needs included wanting to learn about the disease as early as possible, to receive age‐appropriate information that is followed up as they grow older, to feel like they can talk about HD on a day‐to‐day basis, if needed, and to feel like they are in control of the information that is shared with others on their behalf. Second, the findings also suggest that a variety of dilemmas seem to follow the many conversations caregivers have with offspring about HD, including when to talk, what to say, how often the disease should be addressed on a day‐to‐day basis and whom outside the family should be told.

The offspring and caregivers' perceptions of many of the topics that needed to be addressed seemed to mirror one another and reflect something basic in a parent–child relationship: the child's need for knowledge so that the child can make sense of the situation and the dilemmas that arise when parents want to protect the child from the harshness of their life. The general needs the offspring had to talk about regarding the many aspects that came with growing up with a caregiver with HD that corresponds well with findings from past research. For instance, the fact that the offspring wanted to learn about disease from an early age has been well documented when it comes to HD, genetic illness in general, and a range of other diseases, including cancer and HIV (e.g., Dalton et al., 2019; Forrest Keenan et al., 2007; Rowland & Metcalfe, 2013). The need for offspring to talk about HD as a continuous process that develops over time, rather than a one‐time event, has also been reflected in past studies that highlight the incremental nature of telling offspring about genetic risk with HD and other genetic diseases (e.g., Forrest et al., 2003, 2009; Gaff et al., 2007). Our findings add to this knowledge by showing that these conversations also extended to many other aspects of growing up with a caregiver with HD, in addition to talking about genetic risk. For instance, the offspring had to be provided with explanations for the many progressing changes in the caregiver that were experienced as confusing or even frightening, particularly when the offspring did not understand why they occurred. These findings mirror research on other diseases, such as cancer, by showing that children want to be included in honest conversations about the many ways their lives are affected and that caregivers play an important role in helping offspring create an understanding of HD through a variety of different conversations (Dalton et al., 2019). The findings also show that the many consequences it can have for offspring when they do not have their needs for information and conversation met. As supported by past research, not receiving sufficient information leads to feelings of distress, hinders their ability to cope with the situation (Forrest Keenan et al., 2007), and could result in inaccurate and dysfunctional beliefs about their own responsibility for the changes that occurred (Eklund et al., 2020).

The need to talk about HD expressed by the offspring in the current study may also mirror the developmental needs of any child in any situation. For instance, the need to receive information with follow‐up as they grow older and the need to have caregivers who are available for conversations can reflect how a child's understanding develops over time. With younger children, who have a combination of a growing need to predict the world but a poor understanding of the complexity of disease, early information that is not followed up on or explained in age‐appropriate ways can easily lead to misunderstandings and self‐blame (Schonfeld, 1993). The difficulties those who learned about HD in adolescence described, in contrast to learning about the disease when they were younger, reflect how adolescence can be a time of increased vulnerability for anyone, possibly making it a particularly difficult time to first learn about their caregiver's complex disease (Davidson et al., 2015). Also, with the developmental changes that occur from childhood to adolescence, the need for control over information and interference from others will change; here, a need to establish autonomy and identity can easily come in conflict with an increased need for support because of their caregiver's disease (Davidson et al., 2015).

Another key finding in the current study included the variety of dilemmas that seemed to present a more nuanced and complex picture of the many conversations that offspring and their caregivers have about HD than previously presented by the literature. Whereas past research has suggested that questions about whether, what and how to inform offspring about genetic risk is a dilemma for many caregivers (e.g., Forrest et al., 2003, 2009; Gaff et al., 2007; Klitzman et al., 2007), our findings suggest that when it comes to HD, dilemmas also arise in a range of other topics of conversation and, importantly, for both the caregivers and offspring. For instance, our data show that offspring generally preferred being told as early as possible. At the same time, they were unsure about whether all information about HD should be disclosed at this time, how often the topic of the disease needed to be talked about in their everyday lives and whether it would be helpful to share information about HD with others.

Whereas past research has demonstrated how HD severely disrupts family systems and demands that family members try to cope (Brouwer‐DudokdeWit et al., 2002; Forrest Keenan et al., 2007), a major issue posed by HD on family processes identified in the current study was finding the right balance in the many dilemmas they faced throughout conversations about the disease that would lead to the best outcome for the offspring. Some of the families in our study felt they had been successful at achieving a balance in their conversations about disease, that HD had not defined them, that their family had been a resource for the offspring by helping them cope and adapt and that being open to others had been a support. However, other caregivers and offspring did not feel they had achieved a balance in the dilemmas they encountered, and efforts to protect and shield the one another had instead left the offspring uninformed to make important decisions in their lives, with damaged relations within the family and with a lack of trust in those they felt should have helped them. Drawing on knowledge about other diseases and HD (Dematteo et al., 2010; Forrest et al., 2009; Klitzman et al., 2007), we suggest that the ability to successfully balance dilemmas that arise when talking with offspring could partly be tied to the concept of trust. We are not the first to suggest that trust in relationships impacts conversations about HD. The results of one study by Forrest et al. (2009) suggest that when young people first find out about their family history of HD, the trust they have in those who provide the information may impact how the message is received. In another study that looked at patterns of decision‐making when disclosing genetic risk information within families, the concept of trust was described as a ‘gift’ that has the power to strengthen or break relationships when disclosing information more generally (Klitzman et al., 2007). The present study elaborates on these findings by suggesting that trust in relationships could also be relevant in the many conversations that arise about HD, in addition to the disclosure of genetic risk information. For instance, our findings suggest that decisions about *when* and *what* offspring were told seemed to revolve around whether the caregivers trusted their child's ability to understand and cope with information about the disease, and whether the child felt they had trusted that their caregivers' decisions were made in their best interest. Talking about HD on a day‐to‐day basis also triggered issues of trust in the caregiver–child relationship. For the offspring, the actual frequency of the conversations they had seemed secondary to them trusting that a caregiver was available, if needed. The caregivers, on the other hand, needed to trust their ability to handle difficult conversations involving HD and correctly read and interpret their children's evolving needs.

Few studies have explicitly focused on how families affected by complex heritable conditions, such as HD, talk with offspring about the situations they face in their daily lives. The new knowledge brought forth by the current study, which highlights the many dilemmas that can follow conversations about disease and how it impacts the lives of offspring in detail, provides a better understanding of caregivers' and offspring's perspectives on living with HD and directions for areas where these families may need support from health care professionals in the future.

### 4.1 Implications for practice

One of the major objectives for health care professionals, such as genetic counselors, when meeting caregivers affected by disease is to facilitate family communication. However, recent research suggests that there is uncertainty among genetic counselors about how to guide parents to talk with their children (Keenan et al., 2020). The in‐depth insights of the present study can be used to provide clarity and understanding about the many aspects of talking with offspring about HD. The findings demonstrate the need for families to be helped to meet the individual and general needs offspring have for talking about HD and, equally important, help families prepare for and navigate the many and complex dilemmas they may face during these conversations. We suggest that the caregivers in families with HD should be helped to strengthen their ability to make conscious choices regarding current and future conversations with their offspring. We also suggest that families should be helped develop trust in their caregiver–child relationships to feel confident when exploring and addressing their child's individual and developmental needs for conversation about disease. Also, given that offspring in this and past studies indicate that they do not get the emotional support they need from their caregivers, referring offspring to available patient organizations for support could be beneficial and should be considered when assessing the family's needs.

### 4.2 Strengths and limitations

A major strength of the present study is the inclusion of the different perspectives of both caregivers and offspring, reflecting both current and past experiences. Although the experiences recounted by the older participants did not necessarily reflect contemporary views on openness and communication about disease, our participants consistently addressed similar topics, regardless of age. Research has suggested that attitudes and reluctance towards informing offspring in families with HD about the genetic risk have remained stable, despite current possibilities to test for the genetic mutation before symptoms occur (Pierron et al., 2020). As such, we believe this inclusion provided a unique benefit of investigating participants' experiences of conversations about HD throughout childhood and across time. The nature of the sampling in the current study may present some limitations. For instance, it is possible that the offspring who wished to be part of the present study had more difficult experiences they wanted to share, whereas those offspring with few negative experiences may not have felt the same need to participate. In contrast, it is also possible that caregivers who wanted to share their experiences could be those who felt comfortable and confident in their actions and choices regarding their children. Although the current study focused on the perspectives of offspring with a caregiver with HD and caregivers with a partner with HD, we did not include the perspectives of caregivers at risk, presymptomatic, or diagnosed with HD. Because this group is also an integral part of family experiences, future studies could benefit from including this perspective.

## 5 CONCLUSION

The current study has provided a detailed and in‐depth exploration of both caregivers' and offspring's perspectives on talking with offspring about HD. We found that the offspring generally wanted age‐appropriate conversations about the disease as early as possible, which are followed up as they grow older, to have caregivers who are available to talk to on a day‐to‐day basis and to have control over the information that is shared with others about the disease. However, we also found that talking with the offspring about HD included a range of dilemmas that seemed to arise throughout a variety of conversations about the disease, including when to tell, what to say, how often the topic should be brought up in day‐to‐day life and who to tell outside the family. A difficult task for many families appeared to be finding a balance in these dilemmas that would lead the offspring to adapt to and cope with the many challenges presented by having HD in their lives. Our study illustrates the importance of genetic counselors and other health care professionals to help families with HD prepare for and navigate these dilemmas so that knowledge and conversations can be used to strengthen the offspring's ability to adapt to and cope with their caregiver's disease.

## AUTHOR CONTRIBUTIONS

**Siri Kjoelaas:** Conceptualization; data curation; formal analysis; investigation; methodology; project administration; validation; writing – original draft; writing – review and editing. **Tine Jensen:** Formal analysis; methodology; supervision; validation; visualization; writing – review and editing. **Kristin Feragen:** Conceptualization; data curation; formal analysis; funding acquisition; investigation; methodology; project administration; resources; supervision; validation; visualization; writing – review and editing. Authors SK, TKJ and KBF confirm that they had full access to all the data in the study and take responsibility for the integrity of the data and accuracy of the data analysis. All of the authors gave final approval of this version to be published and agreed to be accountable for all aspects of the work in ensuring that questions related to the accuracy or integrity of any part of the work were appropriately investigated and resolved.

## COMPLIANCE WITH ETHICAL STANDARDS

### Conflict of interest

The authors SK, TKJ, and KBF declare that they have no conflicts of interest.

### Human studies and informed consent

Approval to conduct this human subjects research was obtained by the Regional Committee for Medical Research Ethics [Health region East, Norway, reference number: 2017/864347].

### Animal studies

No non‐human animal studies were carried out by the authors of this article.

### Data sharing and data accessibility

Because of the nature of this research, the participants in this study did not agree that their data would be shared publicly; therefore, supporting data are not available.
